# My battle with cancer. Part 1

**DOI:** 10.18632/oncoscience.593

**Published:** 2024-01-03

**Authors:** Mikhail V. Blagosklonny

**Affiliations:** ^1^Roswell Park Comprehensive Cancer Center, Buffalo, NY 14263, USA

**Keywords:** lung cancer, brain metastases, capmatinib, resistance, MET

## Abstract

In January 2023, diagnosed with numerous metastases of lung cancer in my brain, I felt that I must accomplish a mission. If everything happens for a reason, my cancer, in particular, I must find out how metastatic cancer can be treated with curative intent. This is my mission now, and the reason I was ever born. In January 2023, I understood the meaning of life, of my life. I was born to write this article. In this article, I argue that monotherapy with targeted drugs, even when used in sequence, cannot cure metastatic cancer. However, preemptive combinations of targeted drugs may, in theory, cure incurable cancer. Also, I share insights on various topics, including rapamycin, an anti-aging drug that can delay but not prevent cancer, through my personal journey.

## INTRODUCTION

On January 12 (my birthday is on January 13) 2023, I was hospitalized at Mass General Hospital in Boston (the most famous hospital in the world) with dysarthria (impairment of speech) and multiple brain metastases of lung cancer. Ironically, a small vague mass in the lung was seen by X-ray in the Summer of 1991, and it had changed very little eight years later. Therefore, it was decided to ignore it. It was ignored by me and anyone else for the next 24 years. As an MD/PhD and professor of oncology, author of 300 articles on cancer progression and therapy, quasi-programmed aging, and its inhibition by rapamycin, I felt invincible and could not believe that cancer could happen to me. (After all, I was taking rapamycin [1, 2] and quit smoking.) Subconsciously, I may have been anxious and suppressed any thoughts of the asymptomatic harmless mass. I never looked at any X-rays until 2023.

In March 2022, I shrugged off the possibility of lung cancer when I was hospitalized with a stroke, resulting in my left side (leg and arm, but the face was luckily spared) paralysis. My account that the mass had been in the lung for three decades made everyone relieved. Instead, all efforts were focused on finding the source of the thromboembolism. Futile. In retrospect, I am now convinced that stroke was caused by lung cancer [3, 4].

After the initial shock of being paralyzed on March 2022, I decided that, if life gave me lemons, I should make lemonade, and I developed a successful recovery strategy (for me and other stroke survivors).

My recovery was spectacular: from wheelchair to swimming to walking (swimming is easier than walking), to going downstairs (after stroke it is more difficult to go downstairs than upstairs) to running. I even learned new motor skills, such as golf. (I will tell recovery strategy later). The Summer and Fall of 2022 was the happiest time in my life. I had the goal, and life had its meaning.

Meanwhile cancer was silently growing in my lung and creating metastases in the brain, which manifested as speech dysarthria (inability to speak clearly) by January 12, 2023.

Why was it so long (more than 3 decades) between the detection of a benign small mass in 1991 and advanced metastatic lung cancer by 2023?

First, lung cancer can develop over 20 years [5]. For example, smoking-related alterations were found in cancers of former smokers who quit smoking 20 years ago [5].

Second, remarkably, I was taking rapamycin, and rapamycin may delay cancer [2], especially when administered shortly after tobacco use in mice [6, 7]. Treatment with rapamycin could be started early in tumorigenesis. Rapamycin slows down progression of pre-malignant and malignant lesions [6, 7]. In addition, rapamycin delays cancer by slowing aging (risk factor of cancer) [1, 8]. Rapamycin may delay cancer but not prevent it completely. In my case, a pre-malignant lesion, which eventually become cancer, can be traced to 1991, long before I started using rapamycin. Although I was taking rapamycin irregularly and at suboptimal schedules for cancer prevention (I will discuss schedules later), cancer progression might have been slowed down [2]. I was not specifically taking rapamycin to delay cancer, but mostly to delay aging and all age-related diseases including cancer [9]. I was the first scientist who proposed (in 2006) that rapamycin is an anti-aging drug that can be used in humans immediately to slow aging, prevent age-related diseases and extend lifespan [10, 11]. It was proposed three years before the first publication that rapamycin extends lifespan in any animal. Remarkably, rapamycin was a prediction of the hyperfunction theory of aging [10]. Rapamycin is increasingly used by thousands of people as an anti-aging drug (off-label) without side effects [12] https://rapamycintherapy.com/.

This “science and life” article is a personal account on numerous scientific advances in cancer and aging research. It will cover new insights into cancer, anti-cancer drug combinations and therapy-driven tumor progression. I will mention selective protection of normal cells from chemotherapy [13] and explain why anti-cancer drugs are also tumor promoters, whether carcinogens can be anti-cancer drugs [14]. I also will discuss aging and age-related diseases that are a continuation of developmental growth [10, 15, 16].

I will start with an unpublished notion on how metastatic cancer could be treated with curative intent. This is very important for me, as a patient, and I will present it first, just in case I will die, unable to finish the article.

Diagnosed with numerous metastases of lung cancer in my brain in January 2023, I felt compelled to accomplish a mission. Among my numerous unfinished writings, I must select and complete the most important ones. If everything happens for a reason, particularly my cancer, I must discover how cancer can be treated with a curative intent. This has become my mission, the reason for my existence. In February 2023, I understood the meaning of life, of my life. I was born to write this article or this book, exactly.

### Provisional summary of Part I (10/02/2023)

The first requirement is standard: To identify the driver mutation and use its selective inhibitor (targeted therapy) to induce regression in both the primary tumor and its metastases. In my case, the driver mutation is METex14. Treatment with capmatinib, a MET inhibitor, led to a dramatic shrinkage of the lung tumor and most, if not all, of the brain metastases. The therapeutic response was indeed spectacular.

Unfortunately, during tumor regression, an invisible progression of resistance occurs. When the cancer cell burden is high, successful monotherapy will inevitably select for pre-existing resistant—and more aggressive—cancer cells during the therapeutic response. To thwart this selection process, even a single resistant cell with a pre-existing mutation should be targeted. This can be achieved through a combination of the inhibitor of the driver mutation (in my case, capmatinib) and anti-resistance drugs, even though the resistance mutation is unknown (a single cell with a resistant mutation is undetectable). Anti-resistance drugs should be added as soon as possible (immediately after the cancer has responded to the anti-driver drug (in my case, capmatinib). I suggest to target the most anticipated mechanisms of resistance. In METex14-driven cancer, the preemptive combo is suggested: Capmatinib plus Afanitib and Cabozantinib (CAC). In EGFR-mutant-driven lung cancer: Osimertinib, Afatinib, Capmatinib (OAC).

Such preventative treatments aren’t employed in current protocols, but they are essential for a curative approach. These combinations cannot improve initial therapeutic response because there are just a few resistant cells. However, preventing resistance should dramatically prolong progression-free survival (PFS) and overall survival. Furthermore, preemptive combinations should be continually adjusted; anti-resistant drugs can be switched out, and these combinations should be employed sequentially.

Furthermore, a driver mutation could operate in tandem with cooperating mutations. In my case, the METex14 driver mutation collaborates with CDK4 and MDM2 amplification and targeting cooperating alterations may be done sequentially. Conversely, substituting alterations (EGFR, HER2, PDGFR, RET, K-RAS, and so on) should be inhibited simultaneously.

A crucial takeaway is that invisible tumor progression can occur even during tumor regression.

Combinations are essential during tumor regression to prevent progression. These combinations may be frequently modified. Since cancer is continuously evolving, therapy must not only evolve in tandem but stay one step ahead of the tumor. Notably, monotherapy with an anti-resistance drug cannot slow tumor growth, thereby showing no efficacy. Only when it’s added to the drug targeting the dominant driver mutation (in this case, MET14ex), will the anti-resistance drug in combination extend progression free and overall survival.

And now I am starting from the beginning …

### Section 1: My cancer responds to capmatinib very well, but it must be temporarily

#### Chapter 1: METex14 is a driver mutation in my lung cancer

A biopsy of my lung tumor and one of the brain metastases (it’s scary to think about a brain metastasis biopsy) revelated Non-Small Cell Lung Cancer (NSCLC), with a MET exon 14 skipping mutations (METex14). This mutation is found in 3% of NSCLC patients [17]. MET is a receptor tyrosine kinase (RTK) for hepatocyte growth factor (HGF). Activating mutations, such as MET exon 14 skipping mutations (METex14), cause the c-Met kinase hyper-activation, leading to cell proliferation, invasion and epithelial-mesenchymal transition (EMT) and metastasis. HGF stimulates robust and sustained METex14 activation and signaling [18].

The MET receptor kinase activates numerous signaling pathways including Ras-Raf-MEK and PI3K-Akt-mTOR. MET-ex14 is constantly hyper-activated and drives proliferation, EMT, malignant behavior, and metastasis, especially brain metastasis. MET-ex14 is nasty. Especially before the clinical approval of highly selective MET inhibitors, MET-ex14 mutation was associated with a poor prognosis in NSCLC [17]. A patient, like me, with multiple brain metastasis driven by MET-ex14, with neurological progression, would not survive more than a couple of months.

I was fortunate. On May 6, 2020, the FDA fast-tracked the approval of capmatinib (Tabrecta) for metastatic NSCLC patients with METex14 mutations. The approval followed on August 10, 2022. https://www.fda.gov/drugs/resources-information-approved-drugs/fda-approves-capmatinib-metastatic-non-small-cell-lung-cancer.

In patients with metastatic NSCLC with confirmed MET exon 14 skipping (MET-ex14), who had not been treated (like me), the overall response rate was 68% with a response duration of 12.6 months [19].

Data on the real-world overall response rate and real-world progression-free survival were even better. In patients treated with first-line capmatinib (like me), the overall response rate was 90.9% systemically and 87.3% intracranially (brain metastasis), with median systemic progression-free survival (PFS) was 14.1 months [20].

Patients with advanced (27% had brain metastases) METex14-positive NSCLC were treated with capmatinib. The objective response rate for treatment-naïve patients, who had not received any prior treatments, to capmatinib was observed to be 68%. In all patients, the median progression-free survival (PFS) was 10.6 months and the median overall survival was 18.2 months. In the treatment-naïve group, the median overall survival was not reached because it exceeded 18.2 months [19].

Treatment with capmatinib is convenient: two 200 mg pills twice a day.

I was fortunate. Until the biopsy revealed MET-ex14 in the lung tumor and brain metastasis, the plan was to start treatment with Whole Brain Radiotherapy (WBRT), given the progression of neurological symptoms and numerous metastases all over the brain. Sadly, according to Mulvenna et al. (2016), whole brain radiotherapy did not yield any survival advantage for patients with non-small cell lung cancer who had brain metastases. Although the overall survival rate remained unchanged, the treatment was associated with significant side effects [21]. WBRT is associated with high rates of cognitive deterioration and detrimental effects on quality of life [22].

I would especially dislike the potential impairment of the intellect and memory caused by Whole Brain Radiotherapy (WBRT). I would be unable to finish this book. And there would be no survival benefits.

Secondly, I am a recent stroke survivor, having experienced a cancer-related stroke on March 15, 2022. Since then, I have almost completely restored my motor skills by creating a new neuronal network to operate the left (previously paralyzed) side of my body. This neuronal network would be vulnerable to damage caused by WBRT. Furthermore, WBRT, by itself, can cause strokes and stroke-like events [23].

Fortunately, instead of WBRT, I am treated with capmatinib, started on February 4, 2023. Treatment with capmatinib caused dramatic regression of my metastatic brain lesions by February 28. No metastasis progressed, and no new metastasis appeared. My lung tumor also decreased in size dramatically, as measured on April 10, 2023.

My ability to speak was recovering rapidly, so I was able to give a video interview in May. I have finished some of my papers [13, 16, 24] and wrote several papers from scratch [2, 25]. For this period (February-April 2023), I acquired peace of mind and the joy of a meaningful life. Time slowed down (never expected this phenomenon) and so much happened in a short time. I continued capmatinib (MET inhibitor) treatment and tolerated the side effects, which developed after two months of treatment.

#### Chapter 2: Homogeneity of true driver mutations

Advanced cancers exhibit pronounced intratumor heterogeneity, accompanied by numerous genetic and epigenetic alterations [5, 26, 27]. Such complexity underscores the challenge of treating advanced and metastatic cancers using targeted or any other therapy. In essence, achieving a complete cure remains elusive, which explains why my treatment with capmatinib is palliative rather than curative.

This chapter delves into the challenge of cancer’s complexity, aiming to shed light on potential avenues for curative intent. It is important to note that by “curative intent,” we refer to controlling the progression of cancer to an extent where a patient may live with cancer but not succumb to it.

Drawing from my personal experience, this narrative brings to focus my cancer diagnosis, offering insights that might be extrapolated to other cases. An initial biopsy of my cancer, dated 01/18/2023, pinpointed METex14 as the primary driver mutation, with no evidence of other driver mutations. (Note: a comprehensive re-analysis (conducted in May 2023) of the same primary tumor biopsy unveiled overexpression of CDK4, PDGFR, FGFR and others).

In alignment with my single mutation (METex14), my Tumor Mutation Burden (TMB) was initially determined to be relatively low, registering at 3 mutations per megabase. A low TMB typically indicates a low number of oncogenic mutations. It was suggested [17], “METex14 occurs mutually exclusively with known driver mutations.” A thorough explanation, contrasting substitutive mutations against cooperating mutations, will be covered later.

My case exemplifies the model delineated by Vogelstein and colleagues [28, 29]. As noted by Reiter et al. (2018), within individual patients a large majority of driver gene mutations are common to all metastases. The driver gene mutations that were not shared by all metastases are unlikely to have functional consequences [29]. In agreement, in patients with EGFR-mutated NSCLC, necropsies revealed homogeneity in clonal mutations, but heterogeneity in passenger subclonal alterations in different metastasis [30]. As summarized by Reiter et al. (2019), The main points are that True driver gene mutation presents in primary tumor and it’s all metastases. “With a single biopsy of a primary tumor, the likelihood of missing a functional driver gene mutation that was present in all metastases was 2.6%” [28]. Accordingly, individual metastatic lesions usually responded concordantly to targeted therapies. Specifically, “when tumor response from a targeted therapy is observed in one metastatic lesion, it is common for all lesions in that patient to respond to the therapy.” [28]. The timing and degree of the response is dependent on a host of factors [28]. In my case, METex14 was the only driver mutation initially (a limited set of genes was investigated) identified in the primary tumor. The same METex14 was the only mutation found in one of the brain metastases (only one brain metastasis was biopsied for obvious reasons). Successful treatment with the MET inhibitor confirmed that METex14 was a driver. Treatment with capmatinib caused shrinkage of the lung primary tumor and most brain metastases. No one metastasis progressed in size. No new metastasis was detected. At first glance, it may seem that inhibition of METex14 alone may control cancer forever so I could write this article indefinitely. Unfortunately, this may be true with exceedingly very low probability.

#### Chapter 3: Almost enviable resistance to targeted monotherapy

Selected by treatment, a single cell with a pre-existing resistance mutation can render an entire tumor drug-resistant. This resistance inevitably develops unless the tumor is too small to contain any cell with resistance mutations. By eliminating non-resistant cells, capmatinib enables the resistant ones to flourish and repopulate the tumor. Acquisition of resistance results from a good therapeutic response: the intended target cells are eradicated and a resistant subclone from a single cell can repopulate the tumor. (Note: Even the most potent and selective targeted drugs cannot eliminate all sensitive cells because a few drug-tolerant and unfortunate “quantum unluck” cells persist, as will be discussed later). Resistant cells should be killed before they multiply. It is easier to kill one cell than to kill all 10 cells out of 10.

In 2000, it came as a surprise that Imitinib (Gleevec, STI-571), the first clinically-approved targeted drug, caused drug resistance [31]. However, this shouldn’t have been surprising. Effective targeted drugs are more likely to cause resistance than chemotherapy does [31]. As I proposed in 2002 in paper entitled “STI-571 must select for drug-resistant cells but ‘no cell breathes fire out of its nostrils like a dragon’”, a cocktail of multiple targeted drugs might address this problem, since a single on-target resistance mutation against the combo is unlikely to exist in nature [31]. As emphasized, ‘Simultaneous therapy with two drugs is much more effective than sequential therapy. Sequential treatment offers no chance of a cure, whereas combination therapy provides some hope of one [32]. I believe the primary goal of combining targeted drugs is to prevent resistance to drugs that target driver mutations. In my case, it’s capmatinib. The role of other drugs in combinations is to prevent resistance to capmatinib.

Mechanisms of resistance include on-target resistance (for example, mutations that prevent a drug from binding to its target) and off-target resistance (e.g., alterations in other drivers). For instance, resistance to MET inhibitors can arise from alterations in EGFR and other growth factor receptors and Ras. I will review the mechanisms of resistance later. It’s worth noting, however, that off-target resistance (e.g., Ras, EGFR) might make the cancer more oncogenic and aggressive, as reviewed in the 2002 Nature Rev Cancer paper [33]. I will return to this topic later.

### Section 2: Combinations to prevent resistance

#### Chapter 4: Invisible progression during visible regression

Therapy-driven tumor progression starts during therapeutic response (tumor regression).

**Figure 1:**
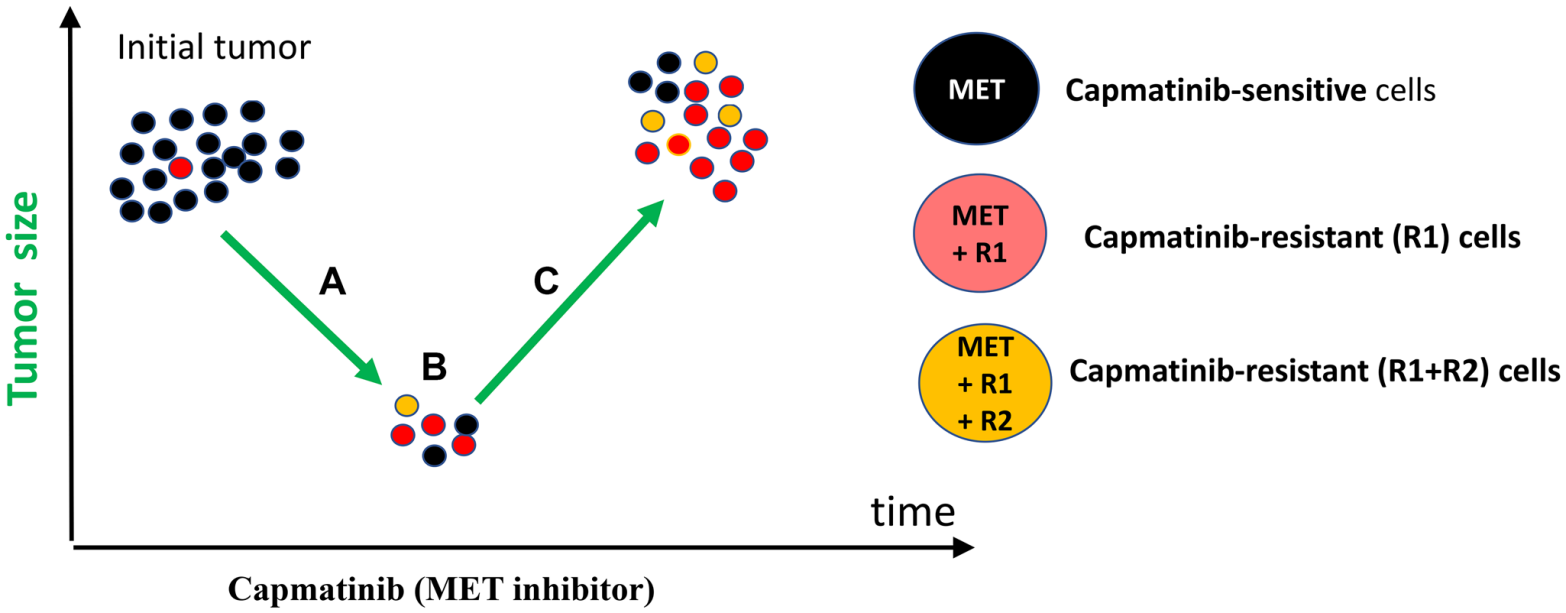
Response-progression round of targeted monotherapy. (**A**) Therapeutic response and tumor regression. Treatment with capmatinib decreases tumor size (radiological regression) by eliminating black (METex14) cells. Red (METex14+R1) cells proliferate (invisible progression) during radiological regression. (**B**) At the lowest point, red cell gain = black cell loss. (**C**) Visible tumor progression. While proliferating, red cells may acquire mutation R2 making them even more resistant (yellow cells).

According to the simplest scenario, all cancer cells have a driver mutation METex14 (Figure 1, black color). One cell also has pre-existing R1 mutation that renders this cell capmatinib (MET inhibitor) resistant (Figure 1). For example, R1 could be activated EGFR, FGF, RET, PDGFR or other GF receptors capable to substitute for METex14. (Note: I am presenting my medical case of MET-driven cancer treated with capmatinib. This information can be extended to any cancer treated with targeted drugs.).

Treatment with capmatinib causes therapeutic response: decrease in cancer cell number and tumor size. However, cancer cells with Resistant (R1) mutation (MET+R1) (Figure 1, red cells) continue proliferation. Their proliferation is exponential [34, 35]. At first, gain of MET+R1 cells is less than loss of MET cells, so a tumor regress radiologically (therapeutic response). It takes expended time (PFS) for cells with pre-exiting R1 mutation to multiply to radiologically significant number, to make progression visible radiologically. (For example, monotherapy with capmatinib for my condition offers PFS of 14.1 months [20]). But invisible progression takes place during therapeutic response: MET+R1-cells are replacing MET-cells (Figure 1). I discuss “resistance progression during response/regression phase” using METex14 (driver mutation) and unknown R1 (for example, activation of K-RAS, EGFR or other GF receptor) because my driver mutation is METex14. The same logic is applicable to any driver (D) mutation in any GF receptors. Some off-target R1 mutations may increase oncogenic potential. So, it may be true progression in oncogenicity.

Also, there is a second source of Resistant (R2) mutations, besides pre-exiting ones. I will call them “proliferation-associated”. Cells generate a few mutations during each division [36, 37]. So, proliferating MET+R1 (Figure 1, red cells) generate random mutations during each round of DNA replication. By chance, one of such R2 mutations can confirm additional resistance to capmatinib. These MET+R1+R2 cells (Figure 1, yellow cells) may be more aggressive and nastier than MET+R1 cells. Although the tumor temporarily regresses in size, it progressed in oncogenicity (Figure 1).

We can formulate crude statements:

Rule 1: The chance of the existence of pre-existent resistance mutation (R1) depends on initial number of cells and mutation burden.
Rule 2: The chance of generating R2 mutations depends on number of replications (proportional to duration of treatment and number of replicating cells) and mutation rate.
Rule 3: Off-target resistance mutations can make cancer cells more oncogenic.

Selection for resistance may leads to a higher oncogenic phenotype [38].

#### Chapter 5: Stable disease

**Figure 2:**
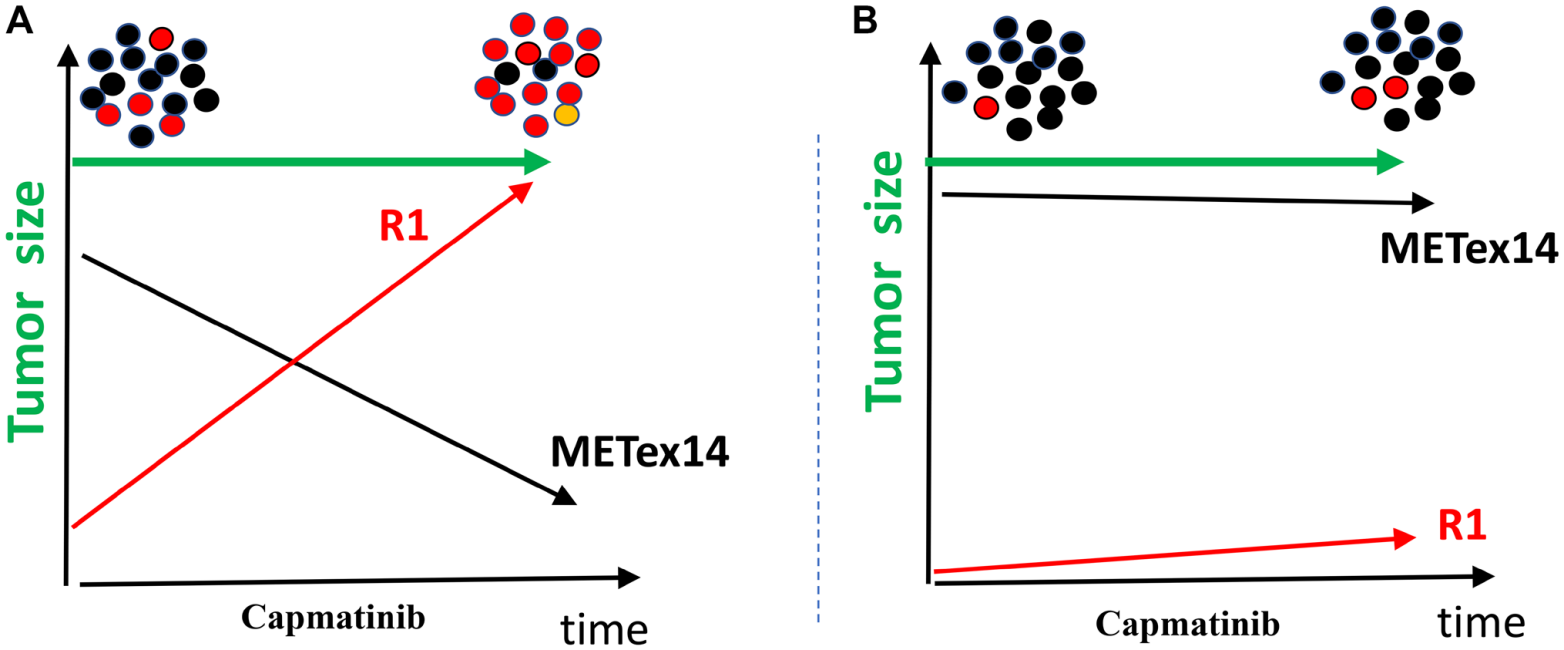
Two models of stable disease. (**A**) Loss of sensitive cells (black METex14) equals the gain of resistant Red (METex14+R1) cells. (**B**) Capmatinib inhibits black cells proliferation but does not kill them. Resistant cells are only a few and proliferate slowly. See Figure 1 for the labels. Green arrow: total cell number, tumor size.

I suggest that stable disease may mask a “progression during regression” (Figure 2A). In cases of stable disease, the treatment does not change the tumor size (Figure 2). It may seem that the drug suppresses proliferation but does not kill cancer cells (Figure 2B). However, why then is stable disease not stable indefinitely? Why does it eventually progress despite continuous treatment? How does cancer become resistant?

According to the “progression during regression” model, while the drug kills sensitive (black) cells, resistant (red) cells replace them (Figure 2A). Initially, the tumor size does not change and remains stable. When most sensitive (black) cells are eliminated, the exponential proliferation of resistant (red) and more deadly cancer cells causes visible tumor progression.

#### Chapter 6: Visible tumor progression ends PFS

Resistant cells grow exponentially during radiological tumor regression. This invisible growth becomes visible disease progression due to the exponential growth of the resistant clone [34, 35]. In my case, tumor regression will also be followed by visible tumor progression, despite continuous treatment with capmatinib, MET inhibitor. Eventually, the increase in resistant (MET+R1) cells will surpass the decrease of sensitive (MET) cells (Figure 1). Invisible oncogenic progression switches to visible growth in tumor size, ending progression-free survival (PFS). Essentially, visible radiological progression is a continuation of the invisible progression observed during regression. The relapsed tumor possesses a different cell composition than the initial tumor (Figure 1) and is more lethal. It is widely accepted that there are fewer treatment options once resistance develops, and it is acknowledged that patients will eventually die.

So, what is next?

#### Chapter 7: Once tumor become resistant the game may be lost

**Figure 3:**
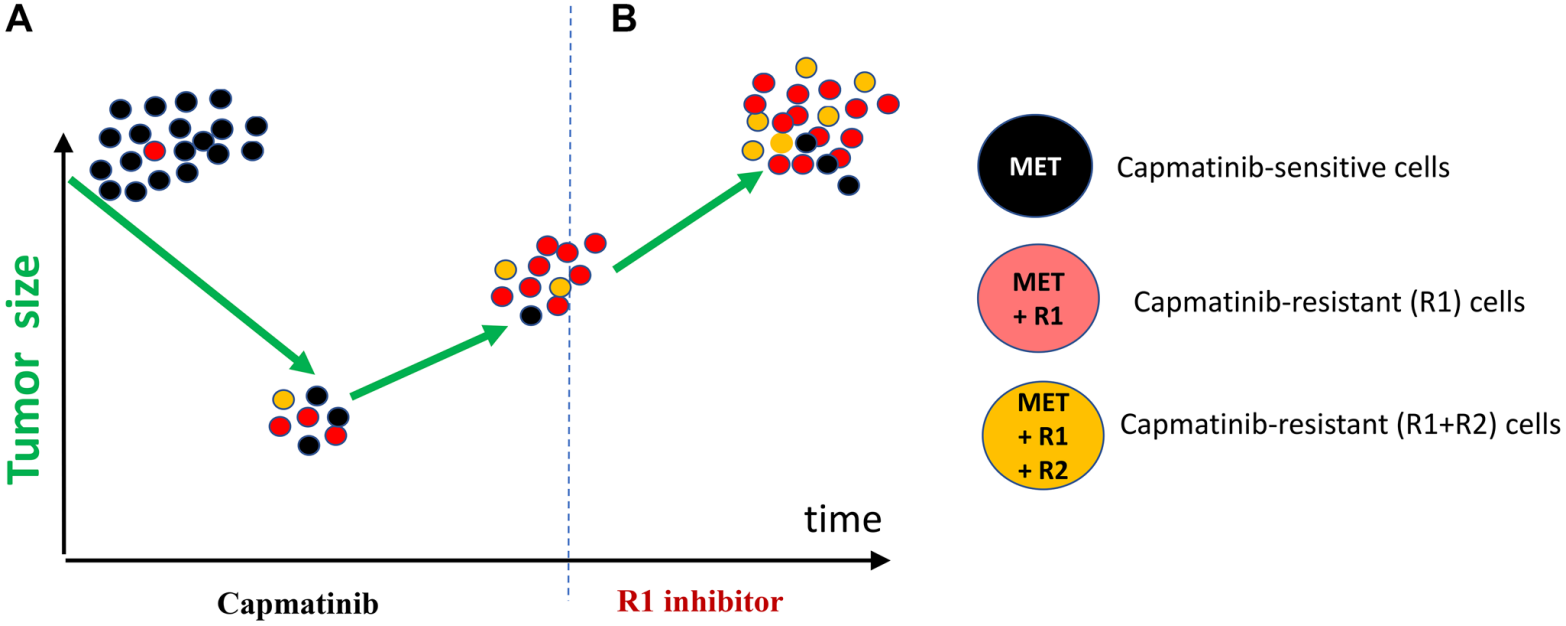
Treatment in sequence is futile (off-target resistance cases). (**A**) Treatment with capmatinib eliminates black cells. (See Figure 1 for the labels). (**B**) Treatment with R1 inhibitor. All types of cells proliferate.

**Figure 4:**
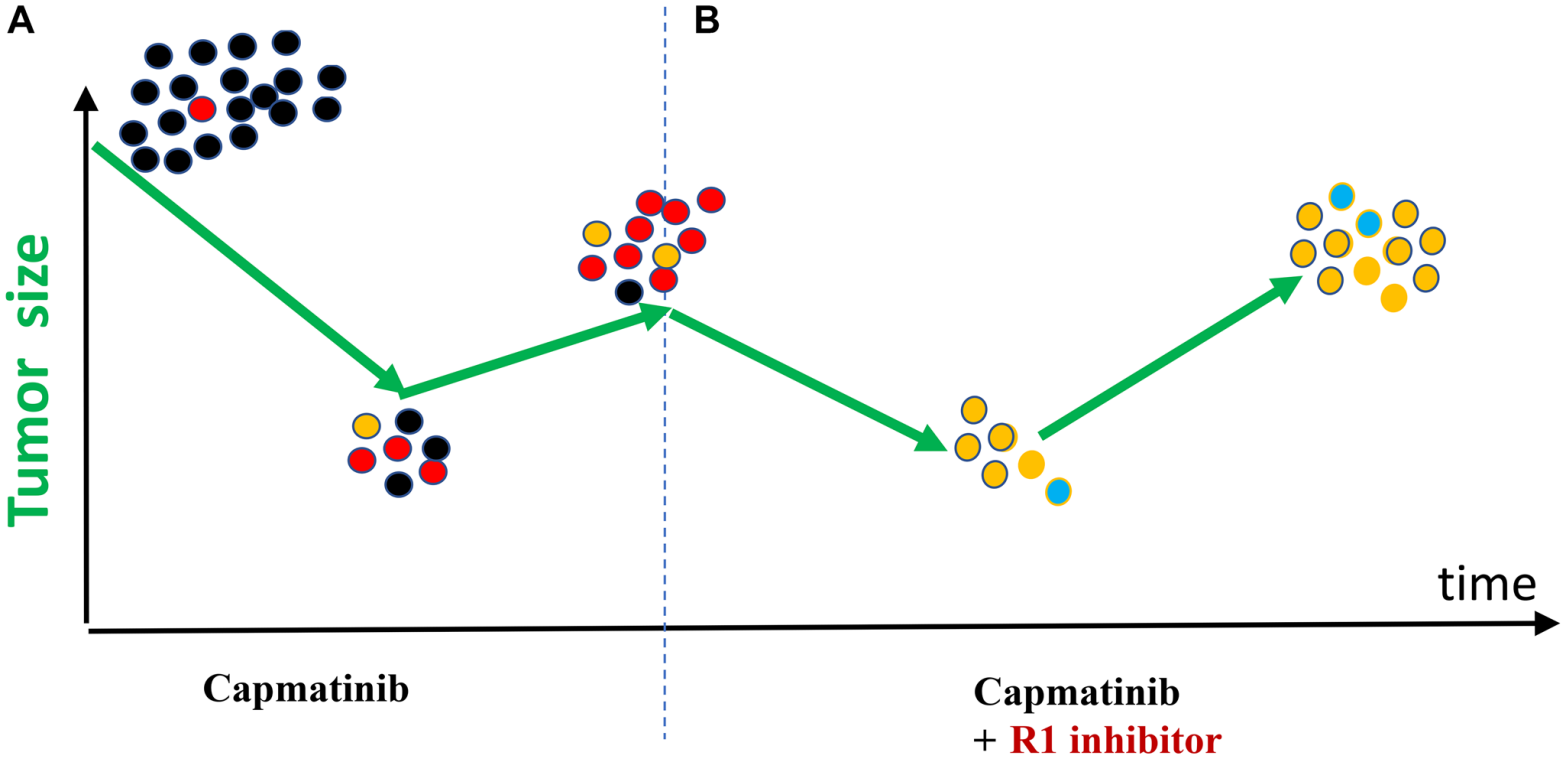
Used after relapse, not from the beginning, combination of capmatinib and R1 inhibitor cannot affect ”yellow” cells. (**A**) Treatment with capmatinib eliminates black cells. (**B**) Treatment with the combination eliminates black and red but not yellow cells (see Figure 1 for the labels). Blue cells are METex14+R1+R2+R3.

Administration of the R1-inhibitor alone, as monotherapy, cannot inhibit tumor growth (Figure 3) because METex14 can still drive tumor growth, mirroring how the initial tumor growth (prior to diagnosis) was driven. Capmatinib alone is no longer effective because all cells already express the R1 mutation in addition to METex14 (Figure 3). R1 can substitute for METex14 in the presence of a MET inhibitor, and METex14 can substitute for R1 in the presence of an R1 inhibitor. Only a combination of capmatinib and the R1 inhibitor suppresses the resistant (Red) cells (Figure 4). Clearly, two drugs are needed to eliminate a cell with two mutant targets that can substitute or replace each other.

While inducing regression, a combination of these two inhibitors (MET and R1) unfortunately cannot cure the relapsed resistant tumor. It might be too late. During monotherapy with capmatinib, resistant “Red” cells proliferate and acquire proliferation-associated mutations, R2 (resistance 2) “Yellow cells”. Figuratively, red cells become yellow (Figure 4). These (METex14+R1+R2) cells resist even a two-drug combination. Treatment of the relapsed tumor with the two-drug combination (Figure 4B) mirrors monotheray of the initial tumor with capmatinib (Figure 4A).

I’ve outlined a model based on my variant of lung cancer, which had the METex14 driver mutation and was treated with capmatinib. This model can be applied to any cancer that possesses a driver mutation and undergoes monotherapy treatment. Sadly, according to current medical practice, monotherapy is administered until the tumor becomes resistant and visibly progress. Only then do oncologists change treatment. And this might be too late. Often targeted therapy is changed for chemotherapy, which usually does not prolong survival at that stage.

#### Chapter 8: Combinations need to be used from the start

**Figure 5:**
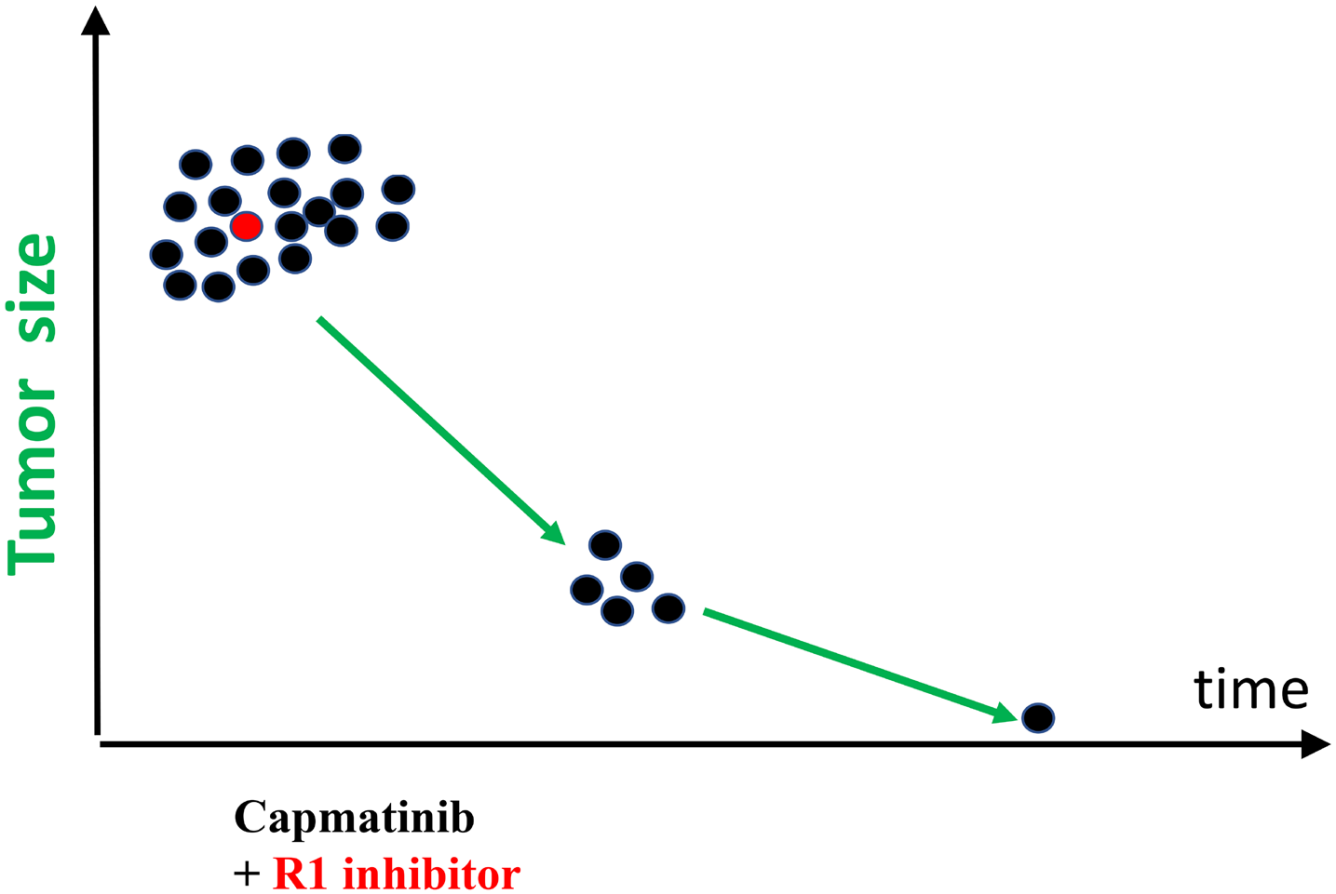
Treatment with the combination may cure cancer. See Figure 1 for the labels.

In theory, combination therapy should be started at the beginning of the treatment (Figure 5). By eliminating just a few resistant cells, combinational therapy may prevent or delay tumor progression and acquiring drug resistance. In contrast, used in sequence drugs fail to control cancer in a long run (Figures 3 and 4). Thus, a combination should be added during regression phase (Figure 5). This conclusion can be based on the pure logic and the simplest model (Figures 1–5).

Using diverse methods, numerous studies reached exactly the same conclusions.

According to Sabnis et al., ideally combinations should be used prior to disease progression [39]. As put by Bozic et al., “combination therapy with two drugs given simultaneously is far more effective than sequential therapy where the drugs are used one after the other”. Furthermore, “sequential treatment offers no chance of a cure, whereas combination therapy offers some hope of a cure [32]. According to Diaz et al. “small number of cells resistant to any targeted agent are always present in large solid tumors at the start of therapy and that these cells clonally expand once therapy is administered. Tumor recurrences are thus a fait accompli when single agents are delivered” [40].

Resistance can be present at the time of initial diagnosis either as a sub-clonal pool of drug-resistant kinase mutations or as co-occurring driver mutations [41]. Starting treatment with a combination of therapies can help prevent the development of resistance from the outset [39]. Resistant mutation is crucial to identify or anticipate early in treatment, as they could quickly limit the efficacy of targeted therapy [39].

### The key word is “anticipated”

**Figure 6:**
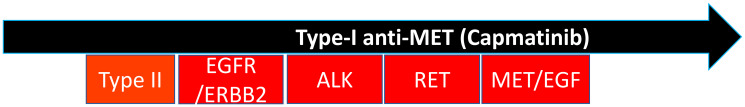
Hypothetical schema: targeting anticipated resistant mutations in sequence. Each drug is combined with capmatinib (black). Red – anticipated resistance alterations. Capmatinib does not generate mutations, it selected for them. So, each mutant pre-exists the treatment with capmatinib.

In my case, anticipated resistance is most likely due to (A: on-target) a secondary mutation in the MET-kinase domain and (B: off-tartet) alterations in EGFR, ERBB2, RET, and other receptor kinases (see Section III). In theory, an inhibitor of the driver, such as capmatinib (in my case), can be combined with one of the inhibitors of anticipated resistance (R1). Treatment with each combination against anticipated resistance should be brief (a month), and then the anti-resistance drug should be replaced by the next one. In my case, anti-resistance drugs should be a type II anti-MET drug (cabozantinib) and an anti-EGFR/ERBB2 drug (afatinib). Their combinations with capmatinib can be used sequentially (Figure 6).

Capmatinib does not generate mutations; it selects for them. Thus, there’s no reason to wait for the multiplication of pre-existing mutations. When the tumor becomes resistant and progresses, we cannot eliminate all these cells.

An anticipated resistance mutation should be targeted before it is detectable by a liquid (or any) biopsy. However, what if the anticipated mutation does not pre-exist and the resistance arises due to an unanticipated mutation/alteration? Still, it is very much worth trying. Just targeting two common anticipated mutations might prevent 50% of resistant outcomes (in my case) (Figure 6). To put it simply, this strategy increases the chances of near-cure from zero to 50%. The difference is profound. It offers to millions of hopeless patients a substantial hope.

#### Chapter 9: Co-therapy with anti-resistant drug should extend remission

In the simplest model, before treatment, all cells contain a driver (D) mutation (in my case, METex14), and one cell contains an additional R1 mutation. R1 renders the cell resistant to D inhibitor (capmatinib, in my case). When expressed in METex14-driven cells, R1 provides no selective advantage in the absence of treatment. METex14 and R1 may even be mutually exclusive mutations. Only when METex14 is inhibited by capmatinib, does R1 substitute (replace) for METex14, and the clone METex14 + R1 starts to grow exponentially during capmatinib-induced tumor regression.

There are several principles:

- The anti-resistance (anti-R1) drug is most effective at preventing resistance, when there is one resistant cell. The chances to kill one cell out of one is much higher than to kill million cells out of million. The later task is practically impossible. So, it is a mistake to wait for tumor progression to start combinatorial therapy.
- The anti-resistance drug alone has no effect on its own and must be used in combination with the anti-D drug (capmatinib, in my case). Anti-R1 drugs would fail in clinical trials.
- Since only one (or a few) R1 cells are present at the beginning of therapy, the combination of two drugs (capmatinib and anti-R1) and monotherapy with capmatinib will produce an equal initial therapeutic response. This could convey a misleading impression of the ineffectiveness of the anti-R1 drug.
- The combination extends PFS (and overall survival) and might potentially lead to a cure.
- In my case, METex14 is the driver mutation, and capmatinib caused fast therapeutic response. I suggest that an anti-resistance drug should be added after this initial tumor repression. If R1 is unknown, anticipated R1 can be determined by statistical data on acquired resistance to MET inhibitors.

### What are anticipated R1s?

On-target alterations (secondary mutations of METex14) comprise 33% of resistance cases [42, 43]. Type I MET inhibitors such as capmatinib selects for resistance mutations, including D1228X and Y1230X. The type II MET inhibitor such as cabozantinib may be used to prevent this type resistance [44]. Resistance mutations against type I were sensitive to type II, and vice versa [45]. Combinations of type I and type II MET inhibitors (for example, capmatinib and merestinib) yielded no resistant clone in cell culture [46]. Cabozantinib, a type II MET inhibitor, caused response in patient with acquired resistant to type-I MET inhibitor [47]. Simultaneous treatment with a type I and type II MET inhibitors may delay the emergence of “on target” MET resistance mutations [46].

I suggest that after capmatinib shows therapeutic effect, it should be temporarily supplemented with cabozantinib. This may eliminate 1/3 of anticipated resistance outcomes.

A common off-target mechanism of resistance involves activation of EGFR, ERBB2/HER2, ERBB4/HER4, KRAS and PI3K pathways [42, 43, 48–50]. Resistance to capmatinib due to amplification of EGFR is common [43, 48, 49]. Dual inhibition of MET and EGFR was proposed to treat resistant METex14 lung cancer [49]. I suggest to combine capmatinib and afatinib (an inhibitor of the EGFR family: EGFR, HER2, and ErbB4). In fact, capmatinib-resistant NSCLC cell lines responded to combination of capmatinib with afatinib [51].

In order to prevent resistance effectively, capmatinib should be combined with drugs that target the most anticipated mechanisms of resistance. Such combinations should be utilized in a sequential manner for brief durations (a few weeks) (refer to Figure 6). This strategy will be discussed in further detail later.

To prevent resistance, capmatinib should be combined with drugs against most anticipated mechanisms of resistance and such combinations used briefly (a few weeks) in sequence (Figure 6). We will discuss that later.

**Regrettably, these proposals have not been implemented in clinical practice. The standard practice involves continuing monotherapy until tumor progression occurs, at which point all cells express R1 resistance. And, in most cases, the game is lost.**

#### Chapter 10: Combinations are the key

The work of Bert Vogelstein and co-workers [28, 29, 32, 37, 40, 52, 53], Razelle Kurzrock and co-workers [54–60] and many other outstanding oncologists convincedly shown that combinations are necessary to prevent resistance.

As stated by Diaz et al. in 2012, a large metastasis contains many cells with different pre-existing mutations conferring resistance to the drug. The time to recurrence is the interval required for the subclone to re-populate the lesion. To make these remissions last longer, combination therapies, targeting at least two different pathways will be required [40].

Immediately after therapeutic response to monotherapy (capmatinib, in my case), its combinations with potential anti-resistance drugs should be used. Sadly, this approach is not used in clinic practice.

As correctly noticed: “The general reluctance to combine medications in oncology may be the exception to the rule in medicine. Indeed, drug combinations are routine in medical practice. Cancer patients, who often have multiple comorbidities, were found to be on polypharmacy.

Therefore, physicians prescribe personalized drug combinations routinely—except in oncology” [55]. “Tumors represent a ‘moving target’ driven by clonal evolution due to therapeutic or time pressure. Innovative combinational therapies early in the course of the disease may help combat the heterogeneity of cancer” [55].

Before I transitioned from cancer research to aging research in 2006, I focused on mechanism-based drug combinations. This predated the era of targeted therapy, so the combinations I explored included both targeted and cytotoxic drugs, aiming to increase the selectivity of the combination [31, 61–67]. Just as a book cannot be written using only one letter, and a picture cannot be created with just one color, cancer should be treated with rationally-designed drug combinations [13, 68].

#### Chapter 11: My opinion on targeted combinations for my cancer

Here I propose “targeting anticipated resistance before tumor progression” or “targeting invisible” resistance before it becomes visible. Crucially, I differentiate between substituting and cooperating alterations.

My emphasis has been on substituting alterations. For instance, EGFR (or other GF receptor kinases) can replace capmatinib-inhibited METex14. This results in the substitution of capmatinib-sensitive cells with resistant ones. As another example, METex14 with the secondary D1228X mutation can replace METex14, driving cancer when native METex14 is targeted. A driver mutation (METex14, in my case) and a substituting alteration R1 should be inhibited concurrently, not sequentially, using a combination. Some substituting mutations might even be mutually exclusive at diagnosis (before therapy).

Conversely, cooperating alterations coexist before therapy and complement each other. These typically involve (a) a driver mutation in K-RAS, or EGFR, or MET, etc and (b) the deactivation of cell cycle brakes, such as the loss of p53, Rb and p16 (an inhibitor of CDK4/6), p53, and Rb, or the overexpression of CDK4 (which inactivates Rb) and MDM2 (which inactivates p53). For example, CDK4 amplification and METex14 cooperate. Cooperating alterations cannot replace each other. Targeting each of them is detrimental to the other oncogenic potential. As such, MET inhibitor (capmatinib) and a CDK4 inhibitor (abemaciclib) should be administered in alternating sequences.

In other scenarios, simultaneous targeting of cooperative alterations is necessary. Consider cancer cells with METex14 and one of resistance mutation R1 (EGFR, HER2, RET, ALK, FGFR, K-RAS) that substitute for MET. Both METex14 and R1 work in tandem with CDK4. Thus, targeting CDK4 impairs R1’s ability to drive cancer growth and act as a substitute for MET. As such, MET inhibitor (capmatinib) and a CDK4 inhibitor (abemaciclib) should be administered together. I will be discussed this later. But I must reveal that I already used these combinations in my treatment. My lung tumor biopsy was re-probed and revealed overexpression of CDK4 (cooperating alteration), overexpression of PDGFR, FGFR (substituting alterations) and unknown RET mutation. All three substituting alteration is targeted by lenvatinib, which also inhibits VEGFR.

**Figure 7:**
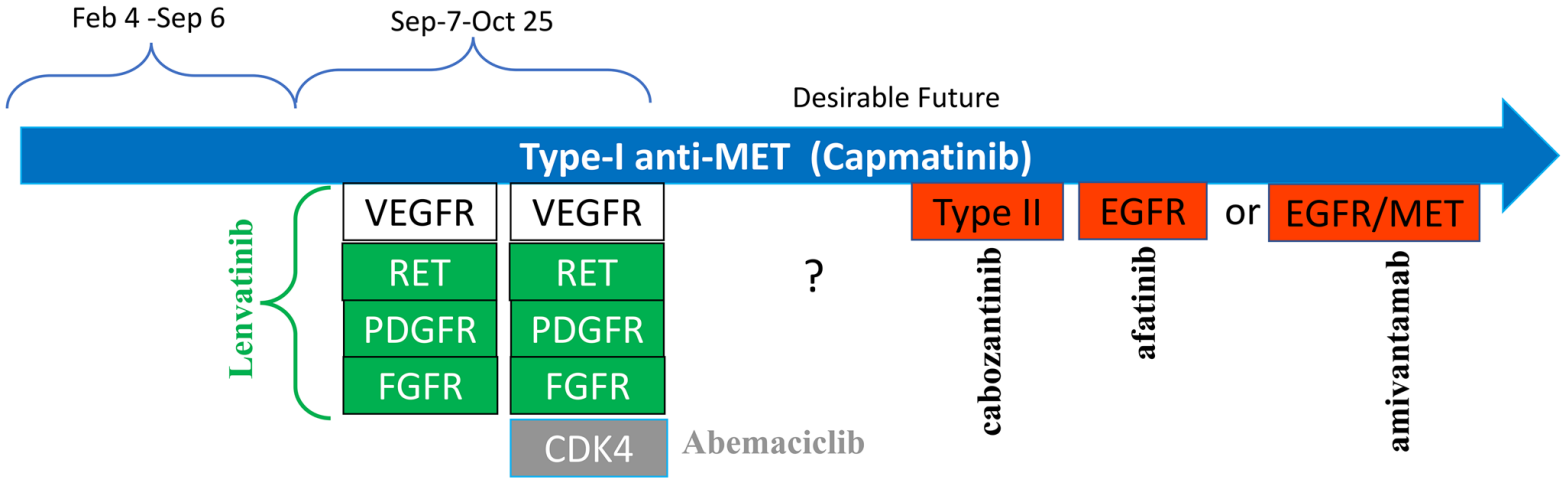
Approximate schema of my treatment: past and desired (by me) future. Green: overexpressed or alerted genes, targets of lenvatinib. Grey: overexpressed CDK4. Red: anticipated resistance alterations.

Combinations include (a) capmatinib + lenvatinib (C+L) and (b) capmatinib + lenvatinib + abemaciclib (C+L+A) (Figure 7). Abemaciclib can also use used alone, for short treatment to allow short holiday from capmatinib, to reduce edema caused by capmatinib.

### Prelude to forthcoming parts

Unfortunately, I was not treated with preemptive combinations. This approach seems too weird to standard oncologists: targeting the invisible, or preventing resistance before this resistance is detectable by any means, the sooner, the better. It may be too late for me, but my book will change the fate of patients in the future.

Supported by Prof. Kurzrock and colleagues, exceptional oncologists and advocates of drug combinations, I received type of combination that I refer to as “co-occurring combo”. This involved targeting co-occurring alterations identified in pre-treatment biopsies (a topic I’ll explore later, comparing preemptive versus co-occurring combinations). My treatment with these co-occurring combinations (Cap+Len and Cap+Len+Abe) prompted an acute therapeutic response, making some brain metastases invisible, but was followed by a similarly rapid progression. It was puzzling. Why is it so rapid development of the resistance to the entire combination in multiple metastasis simultaneously? There is intriguing explanation of this rapid reversal. Based on this explanation, I started a matching treatment. Now, I must pause the book at this remarkable topic. The brain MRI has not been done yet to verify the effect of the treatment. We will know soon. If disease continues to progress, then I could not finish this book. Still, I will post drafts on https://www.mikhailblagosklonny.com/blog/.

### Coming soon

Section III: Resistance to MET-inhibitors: targeting anticipated resistance.

Resistance to EGFR-inhibitors: targeting anticipated resistance.

Preemptive combination to treat EGFR-mutant lung cancer: osimertinib, afatinib, and capmatinib (OAC).

Preemptive combination to treat METex14 lung cancer: captaminib, afatinib, and cabozantinib (CAC).

Section IV: Targeting co-occurring alterations: co-operative alterations and co-drivers. Sequences versus combinations. Combination with inhibitors of angiogenesis.

My treatment with combos: Capmatinib, Lenvatinib, Abemaciclib (CLA).

Section V: Use of radiotherapy for my treatment.

Section VI: Cyclotherapy combinations and their sequence with targeted combinations.

Section VII: Rapamycin for anti-cancer combinations.

Section VIII. Quantum unluck and persistent cells.

Section IX: Targeting non-genetic and normal pathways is cancer cells and normal cells.

Section X: How I should be treated (the ideal scenario and the reality).
